# 原研多光谱智能分析仪诊断肺腺癌浸润程度的诊断性分析

**DOI:** 10.3779/j.issn.1009-3419.2023.101.14

**Published:** 2023-05-20

**Authors:** YANG Xianbei, WANG Peihao, QIN Qi, GUO Kangshun, CUI Yong, LUO Yi

**Affiliations:** 100050 北京，首都医科大学附属北京友谊医院胸外科; Department of Thoracic Surgery, Beijing Friendship Hospital, Capital Medical University, Beijing 100050, China

**Keywords:** 肺肿瘤, 浸润程度, 多光谱分析, 术中冰冻, 神经网络模型, Lung neoplasms, Infiltration degree, Multispectral analysis, Intraoperative frozen, Neural network model

## Abstract

**背景与目的** 肺癌是全世界最常见的恶性肿瘤之一，术中冰冻切片（frozen section, FS）诊断肺腺癌浸润程度的准确率不能完全满足临床需求，本研究旨在探究应用原研多光谱智能分析仪提高FS在肺腺癌中诊断效能的可能性。**方法** 前瞻性采集2021年1月-2022年12月于首都医科大学附属北京友谊医院胸外科行肺结节手术患者的临床资料和多光谱信息，建立神经网络模型并临床验证神经网络诊断模型的准确性。 **结果** 共采集223例标本，最终纳入原发性肺腺癌标本156例，合计1,560组多光谱数据。神经网络模型测试集（前116例的10%）光谱诊断识别组内肺浸润性腺癌和非浸润性腺癌的受试者工作特征曲线下面积（area under the curve, AUC）为0.955（95%CI: 0.909-1.000, P<0.05），诊断准确率为95.69%。临床验证组（后40例）光谱诊断和FS诊断准确率均为67.50%（27/40），二者联合诊断的AUC为0.949（95%CI: 0.878-1.000, P<0.05），准确率为95.00%（38/40）。**结论** 原研多光谱智能分析仪单独诊断肺浸润性腺癌和非浸润性腺癌的准确率与FS相当，应用原研多光谱智能分析仪辅助FS诊断可提高诊断准确率，一定程度上降低术中肺癌手术方案制定的复杂性。

## Abstract (en)

**Background and objective** Lung cancer is one of the most common malignant tumors in the world. The accuracy of intraoperative frozen section (FS) in the diagnosis of lung adenocarcinoma infiltration cannot fully meet the clinical needs. The aim of this study is to explore the possibility of improving the diagnostic efficiency of FS in lung adenocarcinoma by using the original multi-spectral intelligent analyzer. **Methods** Patients with pulmonary nodules who underwent surgery in the Department of Thoracic Surgery, Beijing Friendship Hospital, Capital Medical University from January 2021 to December 2022 were included in the study. The multispectral information of pulmonary nodule tissues and surrounding normal tissues were collected. A neural network model was established and the accuracy of the neural network diagnostic model was verified clinically. **Results** A total of 223 samples were collected in this study, 156 samples of primary lung adenocarcinoma were finally included, and a total of 1,560 sets of multispectral data were collected. The area under the curve (AUC) of spectral diagnosis in the test set (10% of the first 116 cases) of the neural network model was 0.955 (95%CI: 0.909-1.000, P<0.05), and the diagnostic accuracy was 95.69%. In the clinical validation group (the last 40 cases), the accuracy of spectral diagnosis and FS diagnosis were both 67.50% (27/40), and the AUC of the combination of the two was 0.949 (95%CI: 0.878-1.000, P<0.05), and the accuracy was 95.00% (38/40). **Conclusion** The accuracy of the original multi-spectral intelligent analyzer in the diagnosis of lung invasive adenocarcinoma and non-invasive adenocarcinoma is equivalent to that of FS. The application of the original multi-spectral intelligent analyzer in the diagnosis of FS can improve the diagnostic accuracy and reduce the complexity of intraoperative lung cancer surgery plan.

肺癌是全世界最常见的恶性肿瘤之一，也是导致癌症相关死亡的主要原因^[1]^。随着早筛查早治疗观念的推广和低剂量计算机断层扫描（computed tomography, CT）筛查的普及，可疑早期肺癌人群也随之增多^[2]^。目前怀疑为肺癌的肺部结节主要通过手术切除^[3]^，肺癌以非小细胞肺癌为主，而肺腺癌是非小细胞肺癌最常见的亚型^[4]^。目前肺腺癌根据浸润程度不同分为不典型腺瘤样增生（atypical adenomatous hyperplasia, AAH）、原位腺癌（adenocarcinoma in situ, AIS）、微浸润性腺癌（microinvasive adenocarcinoma, MIA）和浸润性腺癌（invasive adenocarcinoma, IAC）^[5]^。指南^[3]^认为对于明确诊断为IAC的结节需行标准的肺叶切除加系统性淋巴结清扫；对于非IAC（AAH、AIS、MIA），可仅行肺楔形切除或肺段切除加局限性淋巴结清扫。术前可根据患者年龄、性别、吸烟、肿瘤标志物水平、CT表现等因素预测结节的浸润程度，结合患者基础情况制定初步手术方案。为实现围手术期肺保护的目的，解决过度医疗问题，肺癌早期手术需在保证肿瘤切缘阴性的同时最大程度保留患者肺功能，所以病灶切除后常需要快速病理结果验证当前切缘是否足够，是否需要扩大切除范围^[6]^。

石蜡病理虽为肺癌诊断金标准，但耗时过长，无法用于快速诊断。目前临床依赖于冰冻切片（frozen section, FS）判断病变良恶性、浸润程度及肿瘤边界情况，但有文献^[7,8]^报道FS在辨别肺腺癌浸润程度时的表现不佳，FS与术后石蜡包埋切片诊断的一致率分别为82.7%和63.24%，客观上给临床工作带来诸多疑惑。所以临床工作中迫切需要一种快速准确判断肺腺癌浸润程度的新技术。

组织快速诊断技术一直是医学领域研究热点，其中光谱诊断技术无创、快速、灵敏、易被患者接受，可以从多个角度反映组织信息。光谱信息经由卷积神经网络分析，建立相应的诊断模型，检测出肿瘤特异信息，最终实现组织性质及浸润程度诊断。大量研究^[9⇓⇓⇓⇓-14]^表明拉曼光谱（Raman spectroscopy, RS）、漫反射光谱（diffuse reflectance spectroscopy, DRS）、傅里叶变换红外光谱（Fourie transform infrared spectroscopy, FTIR）以及近红外荧光（near-infrared fluorescence, NIRF）成像技术均可应用于肿瘤性质诊断，在肺癌、乳腺癌、消化系统肿瘤和宫颈癌等领域，验证了它们的诊断价值。

本研究研发了一款多光谱智能分析仪，通过反射光谱和荧光光谱共同诊断肺结节性质。在前期研究中，我们发现肺癌组织特征光谱结合人工智能数据分析，可以快速自动定量区分正常肺组织和肺癌组织，在区分肿瘤的良恶性以及癌症的浸润程度方面准确率也超过了50%。本研究通过肺腺癌光谱数据库建立神经网络诊断模型，证明原研多光谱智能分析仪区分肺IAC和非IAC的可行性，后续通过临床盲法验证原研多光谱智能分析仪联合FS在区分肺IAC和非IAC的准确性。

## 1 资料与方法

### 1.1 一般资料

本研究为单中心研究，在首都医科大学附属北京友谊医院胸外科进行，研究已获得首都医科大学附属北京友谊医院伦理委员会的伦理许可（No.2022-P2-131-01），并且研究过程严格遵守批准的协议执行。研究过程中，所有被纳入的患者及其家属对本研究全部知情同意并且签署知情同意书。纳入于我院胸外科2021年1月-2022年12月行肺叶切除、肺段切除或肺楔形切除的手术患者，收集并记录每位入组患者的临床资料，肿瘤原发灶-淋巴结-转移（tumor-node-metastasis, TNM）分期采用美国癌症联合会（American Joint Committee on Cancer, AJCC）第八版^[15]^。（1）纳入标准：①2021年1月-2022年12月收治的肺结节手术患者；②年龄18岁-80岁，性别不限；③签署知情同意书；④临床资料完整。（2）排除标准：①石蜡病理诊断不明确；②石蜡病理诊断为非肺原发腺癌，例如小细胞肺癌、肺鳞状细胞癌、腺鳞癌、大细胞癌、类癌、转移癌、淋巴瘤等肿瘤；③术前已通过穿刺活检或细胞学等检查明确病理类型；④术前行放化疗、靶向治疗或免疫治疗；⑤石蜡病理诊断符合，但病灶过小，FS无法确定病灶位置；⑥放置时间超过30 min或被固定液处理的标本；⑦研究过程中表示拒绝参加本研究的患者。

### 1.2 研究方法

#### 1.2.1 多光谱分析仪

（1）设备构成：①高灵敏度的多光谱分析仪（自主研发）；②激光光源发生器（光源A）、激光光源发射器（光源B）、普通发光二极管（light emitting diode, LED）光源（光源C）；③光学探头；④光纤（作为光线通路）；⑤步进电信号控制器；⑥集成在便携式计算机的控制系统。实验原型机如图1。（2）工作原理：步进电信号控制器将便携式计算机发送的信号指令转化成对光源的控制，依次快门式开关光源A、B、C，再由光纤将激光和LED白光传导至光学探头，光学探头集成了高精度光学镜头、光源输出光纤和输入光纤，可以将光源产生的光线引导至组织的表面，探头可同时接收组织受激光照射被激发的荧光以及LED光源照射在组织表面的反射光和散射光，再通过光纤传导至多光谱分析仪，高灵敏度的多光谱分析仪将光谱信息数据化后传输到便携式计算机进行储存以便后续分析处理，光谱采集到数据获取时间不超过3 s。光谱诊断前期基础：本研究在前期可行性探究实验中收集97例手术标本光谱信息，获得了良性病变（炎性）、AIS、MIA和IAC的激光荧光光谱归一化指数图（图2A）；获得了良性病变（炎性）、AIS、MIA和IAC的反射光谱归一化指数图（图2B）；我们发现正常肺组织和肺腺癌在反射光谱波长480 nm处差异最明显。

**图1:**
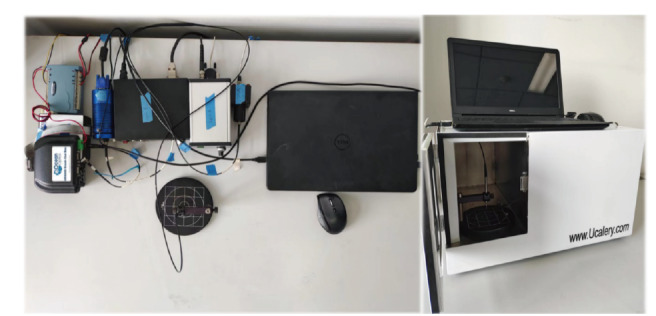
实验用原型机

**图2:**
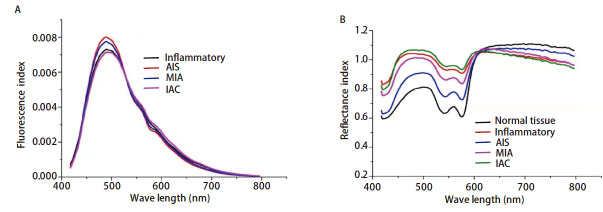
归一化指数图。A：良性病变（炎性）、AIS、MIA和IAC的激光荧光光谱归一化指数图；B：正常组织、良性病变（炎性）、AIS、MIA和IAC的反射光谱归一化指数图。

#### 1.2.2 标准化多光谱采集流程

（1）标本准备：手术标本离体后立即送至病理科（≤5 min），在病理科医生指导下准确找到病灶位置并对半剖开。一半标本立即进行FS处理，另一半标本用无菌纱布轻轻拭去表面血渍和分泌物，显示出病灶及其边界。测量过程中及时拭去测量部位表面血渍和分泌物，保证光学探头紧贴组织表面。（2）光谱测量：遮光条件下在病灶中心及上下左右5个点各测量2组光谱数据（每次测量耗时5 s-10 s），在距病灶边缘2 cm左右随机选取5个点各测量2组数据。每次测量后使用无菌纱布蘸无菌生理盐水擦拭光学探头，保证探头无血渍和分泌物，最后用干燥无菌纱布轻轻拭去表面水渍。最终每个标本会收集包括病灶组织光谱和周围正常组织光谱在内的10组光谱数据，其中每组光谱均包含2份荧光光谱数据和1份反射光谱数据。总测量时间控制在10 min内。

#### 1.2.3 卷积神经网络诊断模型

（1）算法目的：根据输入的光谱数据（1,025个数据点），区分不同组织类型并输出属于某一类的概率值，范围是0-1。（2）建模过程：通过逻辑回归拟合出一个曲面，将这两类分开；采用卷积神经网络来作为拟合架构，我们通过交叉熵损失函数，反向传播梯度值，然后更新卷积神经网络的卷积核参数。本研究将前期光谱数据集中的90%数据作为训练集训练卷积神经网络模型的参数，将剩余10%的数据作为验证集验证训练得到神经网络模型。根据测试集诊断结果调整诊断模型，再重复上述过程。

### 1.3 统计学方法

采用SPSS 26.0软件进行统计分析，连续变量描述为均数±标准差或中位数（四分位范围），根据其分布，使用Student's t检验或Mann-Whitney U检验进行比较。分类变量采用χ^2^检验或Fisher精确检验进行评估。P<0.05为具有统计学差异。诊断结果采用受试者工作特征（receiver operating characteristic, ROC）曲线比较。

## 2 结果

### 2.1 临床及病理特征

在本次研究中，共计采集214例患者的223例标本，患者的纳排情况见图3，病理分布见图4。最终在卷积神经网络诊断模型建立阶段（建模组）纳入116例标本、1,160组光谱数据；卷积神经网络诊断模型临床验证阶段（验证组）纳入40例标本、400组光谱数据，组间患者基线分布无差异，详见表1。

**图3:**
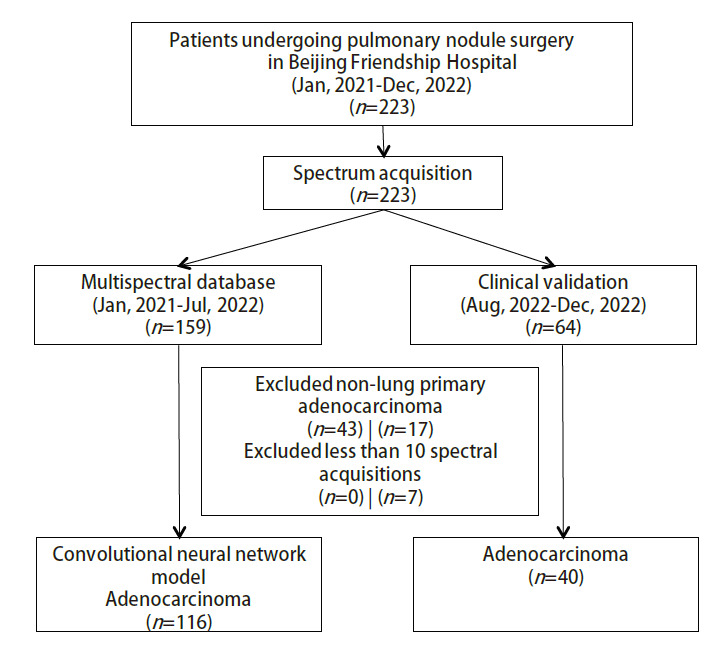
研究流程图

**图4:**
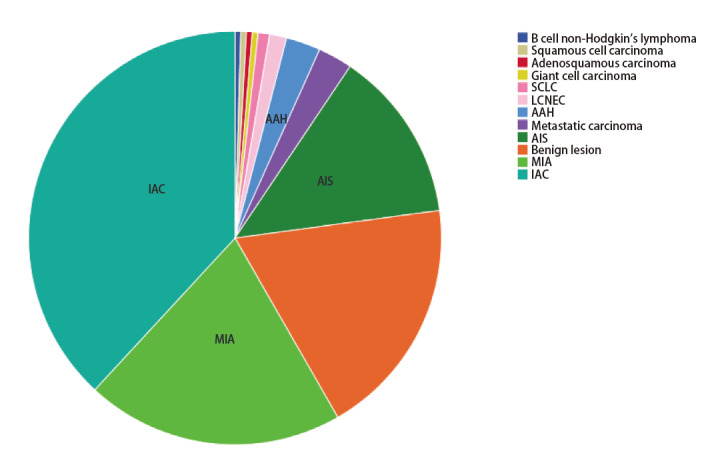
223例患者的病理类型分布

### 2.2 建模组训练集与测试集

共计116份肺腺癌多光谱数据，随机将每例标本10组光谱按9:1的比例纳入训练集和测试集。最终该模型准确区分IAC和非IAC 111例，ROC曲线下面积（area under the curve, AUC）为0.955（95%CI: 0.909-1.000, P<0.05），诊断准确率为95.69%（截断值为0.500），详见图5A。不同截断值的测试结果：截断值为0.344，敏感性为0.937，特异性为0.981，约登指数为0.918；截断值为0.642，敏感性为0.921，特异性为0.981，约登指数为0.902。ROC曲线见图6A。

**图5:**
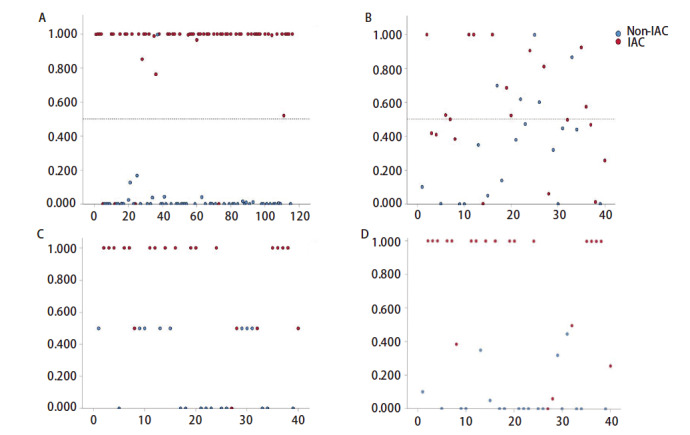
诊断结果散点图（横轴：患者序号，纵轴：浸润指数）。A：建模组（测试集116组）；B ：验证组（单独光谱诊断）；C ：验证组（ FS单独诊断）：FS浸润指数评分：明确浸润=1，明确非浸润=0，不确定=0.5；D ：验证组（ 光谱诊断辅助FS）：辅助诊断定义：FS评分为0.5的标本参考光谱诊断结果计算。

**图6:**
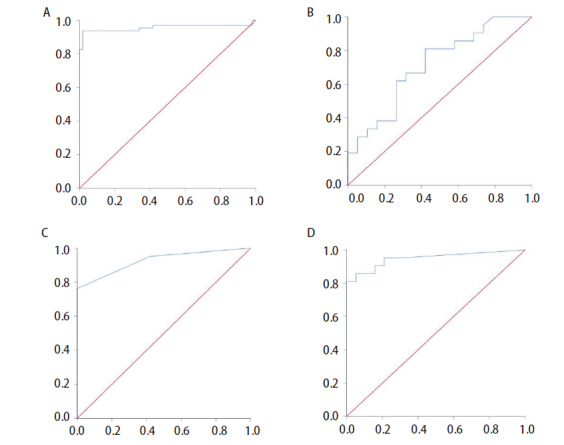
ROC曲线图（横轴：1-特异度，纵轴：敏感度）。A：建模组测试集；B：验证组单独光谱诊断；C：验证组单独FS诊断；D：验证组光谱诊断辅助FS诊断。

**表1:** 建模组和验证组患者基线对比

Item	Modeling group (n=116)	Validation group (n=40)	Z/t/χ^2^	P
Age (yr)	64 (56, 70)	63 (59, 68)	Z=0.020	0.984
Gender			χ^2^=1.727	0.189
Male	39	9		
Female	77	31		
Smoking			χ^2^=0.007	0.932
Yes	21	7		
No	95	33		
Tuberculosis	2	3	χ^2^=3.198	0.074
Chronic obstructive pulmonary disease	1	1	χ^2^=0.630	0.427
Interstitial lung disease	1	0	χ^2^=0.347	0.556
Hypertension	47	16	χ^2^=0.003	0.954
Diabetes	23	5	χ^2^=1.084	0.298
Coronary heart disease	20	5	χ^2^=0.497	0.481
History of cancer	12	7	χ^2^=1.424	0.233
Family history of lung cancer	14	3	χ^2^=0.424	0.639
BMI (kg/m^2^)	24.53±3.30	24.87±2.45	t=-0.593	0.081
BMI grade			χ^2^=1.993	0.737
Low body weight	1	0		
Normal	49	14		
Overweight	45	19		
Mild obesity	19	7		
Moderately obese	2	0		
CEA	10	3	χ^2^=0.049	0.825
CYFRA211	31	12	χ^2^=0.160	0.689
NSE	17	2	χ^2^=2.592	0.107
ProGRP	7	1	χ^2^=0.764	0.382
Pathological tumor diameter (mm)	11 (8, 15)	10 (7, 15)	Z=-0.826	0.409
CT diameter (mm)	12 (10, 18)	13 (10, 19)	Z=-0.299	0.765
CT classification			χ^2^=1.402	0.705
pGGNs	13	6		
mGGNs	73	26		
SN	22	7		
SM	8	1		
Surgical methods			χ^2^=1.676	0.196
Wedge/segmental resection (VATS)	100	31		
Lobectomy (VATS/Open)	16	9		
pT staging			χ^2^=4.266	0.371
T0	2	3		
Tis	21	7		
T1	87	27		
T2	5	3		
T3	1	0		
pN staging			χ^2^=0.559	0.455
N0	113	38		
N1	3	2		
Item	Modeling group (n=116)	Validation group (n=40)	Z/t/χ^2^	P
pM staging			χ^2^=0.347	0.556
M0	115	40		
M1	1	0		
pTNM staging			χ^2^=1.289	0.972
0	23	10		
IA1	35	12		
IA2	39	11		
IA3	9	3		
IB	5	2		
IIB	4	2		
IVA	1	0		

BMI: body mass index; CEA: carcinoembryonic antigen; CYFRA211: cytokeratin 19 fragments; NSE: neuron-specific enolase; ProGRP: pro-gastrin releasing peptide; CT: computed tomography; pGGNs: pure ground glass nodules; mGGNs: mixed ground glass nodules; SN: solid nodule; SM: solid mass; VATS: video-assisted thoracoscopic surgery; pTNM: pathological tumor-node-metastasis.

### 2.3 验证组诊断效能评估

验证组共计纳入40例，最终单独光谱诊断和单独术中冰冻诊断区分IAC和非IAC诊断准确率均为27/40（67.50%）；光谱诊断辅助FS诊断的准确率为38/40（95.00%），其阳性预测值为100.00%，阴性预测值为91.30%，详见图5B-图5D。

### 2.4 ROC曲线

单独光谱诊断的AUC为0.713（95%CI: 0.553-0.873, P<0.05），诊断模型的阳性预测值为68.18%，阴性预测值为66.67%。单独FS诊断的AUC为0.926（95%CI: 0.842-1.000, P<0.05）；光谱诊断辅助FS诊断的AUC为0.949（95%CI: 0.878-1.000, P<0.05），ROC曲线见图6B-图6D。

### 2.5 冰冻诊断不确切标本分析

验证组12例术中冰冻诊断不确定的具体情况见表2。其中石蜡诊断IAC 4例，非IAC 8例。单纯多光谱诊断的准确率为9/12（75.00%），3例低估其浸润程度。

**表2:** 12例冰冻诊断不确切的标本分析

Number	Gender	Age (yr)	Pathological diameter	Infiltration index	Paraffin pathology	Results of intraoperative FS diagnosis
13	Female	68	12	1.0	MIA	Lung AIS with focal infiltration, final paraffin and immunohistochemistry.
15	Female	64	4	0.5	MIA	At least AIS of the lung, visible small focal infiltration, limited frozen sampling and sectioning, and final paraffin.
25	Female	66	8	3.8	IAC	Lung AIS with focal infiltration, final paraffin and immunohistochemistry.
26	Female	59	7	0.0	MIA	At least AIS of the lung, visible small focal infiltration, limited frozen sampling and sectioning, and final paraffin.
27	Female	59	5	0.0	MIA	At least AIS of the lung, visible small focal infiltration, limited frozen sampling and sectioning, and final paraffin.
34	Female	64	4	0.5	AAH	Atypical adenomatoid hyperplasia, AIS with invasion cannot be excluded.
48	Female	68	20	0.6	IAC	Lung AIS with focal infiltration, final paraffin and immunohistochemistry.
49	Female	50	7	3.2	MIA	Lung AIS with focal invasion was considered to be invasive adenocarcinoma and was finally left to paraffin.
50	Female	50	5	0.0	MIA	Lung AIS with focal infiltration, final paraffin and immunohistochemistry.
51	Female	50	14	4.5	MIA	Lung AIS with focal invasion was considered to be invasive adenocarcinoma and was finally left to paraffin.
52	Female	63	10	5.0	IAC	Lung AIS with focal invasion was considered to be invasive adenocarcinoma and was finally left to paraffin.
64	Female	35	9	2.6	IAC	Lung AIS with focal invasion was considered to be invasive adenocarcinoma and was finally left to paraffin.

FS: frozen section.

## 3 讨论

目前快速诊断技术主要有FS、光谱分析技术和质谱分析技术。临床常用的诊断方法为FS。Walts等^[16]^对比224例肺腺癌患者的冰冻结果和石蜡病理结果，发现FS错误和延迟的发生率分别为12.1%（27/224）和6.3%（14/224）；Konno等^[17]^分析96例怀疑恶性的肺结节患者的FS和最终诊断资料，发现2例诊断不一致，14例诊断延迟，FS的整体准确率为83.3%（80/96）。光谱分析技术主要识别组织内的特定结构，如激光荧光光谱通过组织内的特异性荧光基团识别，DRS、RS和红外光谱的诊断主要是通过组织表面和组织内部对光的吸收、散射、反射作用，最终引起入射光和反射光波长差异^[18]^。在本研究前期基础过程中，如图2，归一化反射光谱图显示不同组织的光谱差异显著，尤其是区分正常组织和病变组织的能力突出；归一化荧光光谱图区分炎性组织和浸润性病变的能力欠佳，但有区分不同浸润程度病变的潜力。目前有多项研究使用单光谱区分正常肺组织和肺癌组织，例如Zhang等^[19]^使用表面增强对光谱肺癌和健康肺组织进行分类，其灵敏度和特异度均达到95.7%。Zhang等^[20]^基于高光谱成像技术结合三维卷积神经网络分析处理肺癌病理图片，最终总体平均值为0.962，精确度、召回率和Kappa值均超过0.920。质谱分析是一种与光谱分析并列的谱学方法，通过分离、检测气相离子来鉴定化合物的一种诊断技术。目前应用于组织快速诊断的主要为环境分析质谱：解吸电喷雾电离质谱（desorption electrospray ionization-mass spectrometry, DESI-MS）、快速蒸发电离质谱（rapid evaporative ionization mass spectrometry, REIMS）和基质辅助激光解吸电离质谱（matrix-assisted laser desorption ionization time of flight mass spectrometry, MALDI-TOF-MS）。Ma等^[21]^使用REIMS获取多形性胶质母细胞瘤的脂质谱，用于脂质组学分析和肿瘤实时诊断，最终诊断准确率高达94.74%。另有研究^[22]^使用结合探针电喷雾电离质谱和机器学习模型诊断肝肿瘤，其诊断准确率波动在92.6%-98.3%。总体而言，在组织快速诊断领域，FS、光谱分析技术和质谱分析技术均可准确识别肿瘤组织和正常组织，后两者在组织差别较小的肿瘤亚组分析方面具有潜力。

本研究中，FS诊断肺IAC和肺非IAC的整体准确率为67.5%，与光谱诊断准确率相当，但FS诊断的ROC曲线优于光谱诊断，且FS诊断准确率不高主要是病理诊断不确切的部分占比较高（12/40），在FS诊断明确部分仅有1例错误（1/40）。因而本研究采用光谱诊断技术辅助FS诊断而非FS诊断辅助光谱诊断，辅助过程：同时进行光谱诊断和FS诊断，当FS诊断结果明确时以FS诊断结果为准，当FS诊断不确切时参考光谱诊断结果，最终结果表明光谱诊断技术辅助FS可以显著提高肺IAC和非IAC的诊断准确率，其AUC为0.949（95%CI: 0.878-1.00, P<0.05）。该诊断模型的阳性预测值为100.00%，阴性预测值为91.30%。较高的预测值得益于术中冰冻明确诊断的部分基本与石蜡病理相符，诊断不确切的部分可通过多光谱诊断量化浸润指数评分，进而优化诊断结果。另外，据相关研究报道，肺癌发生的危险因素有年龄、吸烟、二手烟、空气污染、职业暴露、病史、肺部疾病等^[23]^；另有基于CT影像学检查的风险预测模型可以较为准确地预测肺癌^[24]^。Wu等^[25]^回顾性分析291例部分实性肺结节手术患者的临床信息和影像学特征，其构建的侵袭性肺腺癌预测模型与实际基本相符，其准确性达84%。Hu等^[26]^纳入2,018例肺腺癌患者临床特征和影像学特征建立多参数预测模型并验证其诊断IAC的能力，其多参数预测模型的AUC（0.883）高于单模块模型（0.827）或最大CT值模型（0.791）。本多光谱智能分析仪后续可在诊断模型内添加患者肺癌高危因素等基础信息以及术前CT数据，进一步优化诊断模型。

与高光谱成像等光谱分析技术采集彩色图像信息不同，原研多光谱智能分析仪采用三光源发射器，收集新鲜离体组织浅表的反射光谱信息和自体荧光光谱信息，无需标本预处理以及染色切片等操作，单次光谱采集时间约3 s，时效性更强。结果发现不同病理组织的反射光谱归一化指数图差异明显，荧光光谱归一化指数图的差异次之，我们发现通过反射光谱数据即可使肺癌组织和周围正常肺组织的鉴别率接近100%，荧光光谱在鉴别过程中贡献不高。另外Chen等^[27]^研究了RS在肺癌诊断中的整体性能，通过荟萃分析12篇相关研究，发现RS在肺癌中的汇总诊断敏感性和特异性分别为90%（95%CI: 87%-92%, P<0.05）和76%（95%CI: 72%-79%, P<0.05）。由此可见，单一光谱鉴别肺癌与正常组织切实可行，但我们在区分肺腺癌浸润程度过程中发现单一反射光谱区分效果不佳，荧光光谱可作为反射光谱的辅助来提高诊断效能。Lin等^[28]^使用类似的方法，开发了一个集成白光成像、RS、DRS和荧光光谱的四模态内窥镜系统，采用主成分分析和线性判别分析精选数据处理分类；并用该系统进行了鼻咽癌检测的临床实验，其诊断灵敏度和特异度分别为98.6%和95.1%，在体内实时诊断展现出巨大潜力。多光谱诊断系统可以弥补单一光谱在探测深度、空间分辨率、时间分辨率等方面的不足，同时不同光谱（例如RS、DRS和荧光光谱）的诊断原理不同，反映的组织信息内容也不同，多光谱检测可采集更多的组织信息，为卷积神经网络诊断模型提供更有力的支撑。

本研究将肺腺癌划分为IAC和非IAC（AAH、AIS、MIA）。一方面，AAH、AIS、MIA的病变多为早期，组织结构变异不明显；另一方面，上述病变直径较小，光谱分析仪（探针直径2 mm）采集区域可能有重叠，或被病变周围正常组织干扰。由于IAC预后显著劣于AAH、AIS和MIA，我们在二者之间划线，以期精准识别危害程度更高的浸润性病变。结果显示光谱诊断单独区分二者的准确率为67.50%，与建模组的准确率差异较大。一方面是建模过程多次调试诊断模型，本研究采用了拟合度最高的诊断模型，可明确提高建模组诊断准确率；另一方面是建模组训练集的识别率可达100%，训练集和测试集数据均来自同一批患者的不同数据，不同数据在采集过程中可能存在重叠，这将导致建模组诊断准确率偏高。后续研究可通过减少采样重叠率、改进光学探头等方式减小误差。

光谱技术作为经典的组织快速诊断技术，在多种疾病诊断中均有应用。本研究创新性整合反射光谱、荧光光谱等多种光谱，配合卷积神经网络模型分析，能够最大程度地利用光谱信息，利用其组织鉴别能力加以区分IAC和非IAC。首先，我们充分利用人工智能诊断的量化优势，结合FS共同诊断，降低FS诊断的不确定率，并最终提高IAC诊断准确率；其次，我们使用深度学习模型优化现有诊断模型，随着多光谱数据累计，其诊断准确率愈发接近石蜡病理。

本研究的局限性：（1）本研究为单中心研究，受限于样本量原因，多光谱分析诊断模型的独立诊断准确率不高，后续可通过开展多中心研究，扩大样本量，积累足够多的光谱数据，进一步提高准确率。（2）未纳入患者一般资料和CT影像学资料共同用于建立诊断模型，后续研究计划将三者共同用于IAC的诊断。（3）研究过程中发现部分送达时间过晚（>30 min）标本的正常组织和病变组织无法区分，标本肉眼观察呈红黑色，或与组织缺血缺氧时间过长有关。本研究虽然严格控制标本离体送达时间，但从第一组光谱数据采集开始到结束仍花费约6 min，后续会优化实验流程，尽可能缩短光谱采集时间。（4）研究中未被纳入分析的其他病理类型的标本如肺鳞癌、肺腺鳞癌、肺大细胞神经内分泌癌、大细胞癌、转移癌等恶性肿瘤的样本数量较少。该光谱诊断系统理论上具有鉴别区分上述病变的潜力，因此我们同样需要进一步扩大其他病理类型的标本数量和种类，以探索该系统对其他肿瘤的诊断能力。

光谱技术和光谱分析正在朝着数字化、智能化、网络化不断发展，基于其定量诊断的优势，未来结合放射组学相关研究，其在快速诊断领域的地位会愈发重要。当前多光谱技术具有无创、快速、灵敏、准确、便携和经济等特点，结合本研究结论，多光谱诊断可用于辅助术中冰冻技术用于肺癌，作为冰冻诊断不确切时的补充诊断，还可推广至乳腺癌和甲状腺癌等需要术中快速诊断以明确手术范围的患者。此外，多光谱诊断有希望识别转移性淋巴结，将来或可用于术中前哨淋巴结检查，甚至于常规淋巴结活检。光谱检查多为探针式，将来可通过集成至活检针上进行多种癌症的原位诊断，进一步减少等待时间。

综上所述，光谱诊断和FS诊断准确率相当，但FS整体诊断效能优于光谱诊断，光谱诊断辅助FS诊断可显著提高鉴别肺IAC与非IAC的能力，术中对于FS诊断不确定的患者，可考虑通过光谱诊断进行补充，指导手术决策。但鉴于本研究为单中心研究，且样本量较小，故仍需进一步前瞻性、多中心研究验证。
